# *Don’t change who we are but give us a chance:* confronting the potential of community health worker certification for workforce recognition and exclusion

**DOI:** 10.1186/s13690-022-00815-4

**Published:** 2022-02-21

**Authors:** Ashley Kissinger, Shakira Cordova, Ann Keller, Jane Mauldon, Lori Copan, Claire Snell Rood

**Affiliations:** 1 Center for Healthy Communities, Environmental Health Investigations Branch, California Department of Public Health, 850 Marina Bay Parkway, Building P-3, CA 94804 Richmond, USA; 2 School of Public Health, University of California, Berkeley, CA USA; 3 Goldman School of Public Policy, University of California, Berkeley, CA USA

**Keywords:** Community health workers, *Promotores de salud*, Community health, Certification, Workforce development, Qualitative methods

## Abstract

**Background:**

For community health workers (CHWs) and *promotores de salud* (CHWs who primarily serve Latinx communities and are grounded in a social, rather than a clinical model of care)*,* the process of certification highlights the tension between developing a certified workforce with formal requirements (i.e., certified CHWs) and valuing CHWs, without formal requirements, based on their roles, knowledge, and being part of the communities where they live and work (i.e., non-certified CHWs). California serves as an ideal case study to examine how these two paths can coexist. California’s CHW workforce represents distinct ideologies of care (e.g., clinical CHWs, community-based CHWs, and *promotores de salud*) and California stakeholders have debated certification for nearly twenty years but have not implemented such processes.

**Methods:**

We employed purposive sampling to interview 108 stakeholders (i.e., 66 CHWs, 11 program managers, and 31 system-level participants) to understand their perspectives on the opportunities and risks that certification may raise for CHWs and the communities they serve. We conducted focus groups with CHWs, interviews with program managers and system-level participants, and observations of public forums that discussed CHW workforce issues. We used a thematic analysis approach to identify, analyze, and report themes.

**Results:**

Some CHW participants supported inclusive certification training opportunities while others feared that certification might erode their identity and undermine their work in communities. Some program managers and system-level participants acknowledged the opportunities of certification but also expressed concerns that certification may distance CHWs from their communities. Program managers and system-level participants also highlighted that certification may not address all challenges related to integrating CHWs into health care systems. CHWs, program managers, and system-level participants agreed that CHWs should be involved in certification discussions and decision making.

**Conclusions:**

To address participant concerns, our findings recommend California stakeholders build a voluntary certification process structured with multiple pathways to overcome entry barriers of traditional certification processes, maintain CHW identity, and protect diversity within the workforce. Positioning CHWs as decision makers will be critical when designing state certification processes.

## Background

Community health workers (CHWs), including *promotores de salud,* are trusted members of the community who have an intimate understanding of the population they serve [1–6]. *Promotores de salud* are a subset of CHWs who primarily serve Latinx communities and are grounded in a social, rather than a clinical model of care [7, 8]. Their community membership, language, and cultural relationships allow CHWs and *promotores de salud* to bridge health care and social services divides within the current population health service system, facilitating the delivery of health promotion and culturally informed interventions [1–6]. CHWs build individual and community capacity by increasing health knowledge and self-sufficiency through conducting outreach, providing education, connecting communities to social support services, and advocacy [9]. CHWs may be employed by a clinic, hospital, health department, or community-based organization, or as volunteers [10, 11].

In most states, CHWs and *promotores de salud* work outside of any system of formal certification. Nationally, there are no unified training standards for CHWs [12]. However, there are nationally recognized CHW skills and competencies [13]. Approximately 20 states have developed certification standards [14]; these are intended to enhance the credibility of qualifying professionals, increase service quality, and assure those served of CHW competency [15–18]. Statewide CHW certification involves developing a standardized process for documenting the proficiency of individuals across the state in the core skills and roles of a CHW [17]. Training alone does not lead to certification. Certification processes identify requirements related to CHW training or experience, formal education, language, and criminal background and are usually overseen by a certifying agency (i.e., state, educational institution, or private entity) that administers a competency-based examination to certify individuals with the skills and knowledge necessary to perform relevant tasks [17, 19, 20]. Certification differs from an educational certification of completion and also credentialing or licensing, a legislative directive where an individual must obtain a credential or license to practice or work under that job title [19].

A key policy question is whether and how a state should introduce certification for CHWs [21, 22]. The CHW workforce stands at a crossroads: to advocate for certification or oppose it. One path leads to a certified workforce integrated into health care systems via formalized training and qualifications [23]. The other path retains the current emphasis on CHWs as part of the communities where they work, valued for their community relationships [23]. As there is very limited evidence about how certification impacts the workforce [24], we cannot say with certainty that certification standardizes the CHW workforce or maintains CHWs’ community connection.

Importantly, CHWs historically have not led their own workforce development, possibly because they are often from marginalized communities and are economically vulnerable [10, 19, 25]. This is now changing. CHWs have been involved in developing certification in some states (e.g., Arizona and Massachusetts) and have led national CHW efforts (e.g., National Association of Community Health Workers) [26–29].

This study explores the debate through data from California, presenting diverse CHW stakeholder perspectives on CHW certification. As in many states, California’s CHW workforce includes CHWs with diverse roles and training, who represent distinct ideologies of care [7, 8]. Clinical CHWs are most often employed by clinics and hospitals. Community-based CHWs work for health departments and community-based organizations. *Promotores de salud* are typically volunteers and provide services to primarily Latinx communities through a social rather than a clinical model of care [7]. Most recently, California’s managed care organizations have started employing CHWs to provide health promotion services [20, 30]. This study is relevant for other states considering certification to address their CHW workforce and poses a certification option that may preserve CHW identity while also protecting diversity within the workforce.

## Methods

We aimed to explore perceptions among diverse stakeholders about possible benefits and consequences to implementing CHW certification in California. The lead author employed a purposive sampling approach to recruit three categories of stakeholders (all from California): 1) CHWs (part-time, full-time, and volunteer), who could be clinical CHWs, community-based CHWs, or *promotores de salud*, 2) managers of programs that employ CHWs, and 3) system-level participants, such as employers, coalition leaders, academic researchers, foundations, policymakers, and health officers. They represented various organization types (e.g., hospital, community-based organization, government, clinics, managed care organizations, advocacy organizations, academia), and communities served. We also recruited experts from the federal government, national coalitions, and organizations with technical expertise in CHWs and certification. The lead author identified experts if they had provided technical assistance to states implementing certification, had publications related to CHW workforce development, including certification, or led CHW workforce initiatives (e.g., taskforces). All participants were recruited by phone and email and provided verbal consent to participate in the study. Participants were offered up to $100 compensation (for up to three-hour participation in focus group or interview, travel time, and childcare expenses), which was not always accepted. The study was approved by the University of California Berkeley Committee for Protection of Human Subjects.

We used focus groups, interviews, and observation of public forums to gather diverse CHW stakeholder perspectives on CHW certification in California. The lead author conducted focus groups primarily with CHWs to allow for discussion among workforce members. The lead author conducted interviews with program managers and system-level participants to obtain in-depth contextual information on California’s CHW workforce in a manner that was feasible with their time constraints. Focus groups and interviews were semi-structured with a single guide that sought input and recommendations on CHW certification using examples of certification programs (one voluntary, one required) from two other states (i.e., Texas and Massachusetts). During focus groups and interviews, participants received an overview of certification and how it differs from credentialing or licensing. Focus groups lasted up to three hours, and interviews lasted approximately one hour and were audio recorded and transcribed. Focus groups and interviews were conducted in either English or Spanish.

From October 2018 – November 2019, the lead author conducted 44 focus groups, group interviews, and individual interviews, with 66 CHWs, 11 program managers, and 31 system-level participants. Focus groups ranged from three to nine participants. The lead author conducted predominantly individual (i.e. one-on-one) interviews with program managers and system-level participants, but in some cases, program managers and system-level participants requested group interviews (two to three people) when both worked at the same organization. Focus groups were conducted with CHWs to facilitate discussion across workforce peers. Most of the focus group and interviews were conducted in English, while six focus groups were conducted in Spanish. All focus groups and interviews were conducted at the organization where stakeholders worked. Nearly all interviews with system-level participants were conducted in person, although some were conducted by phone when in-person interviews were not feasible. In addition, we conducted ten observations in public forums (e.g., conferences, taskforce meetings, coalition meetings) where CHW workforce issues, including certification, were discussed. Observations offered context to how stakeholders leveraged CHW work and their plans to apply CHW certification in real-world settings that may not have been discussed in individual interviews or focus groups. The lead author took detailed field notes to document how CHW workforce issues were discussed, the context in which certification was described.

We used a thematic analysis approach to identify, analyze, and report patterns or themes within the focus group, interview, and observation data [31]. All focus group and interview recordings were transcribed in their original languages. Spanish-language focus groups were transcribed into Spanish and then professionally translated and checked by the Spanish-speaking authors for accuracy. We reviewed the focus group and interview transcripts and observation field notes to identify preliminary analytic categories. The two Spanish-speaking authors developed the codebook through inductive coding and analyzed the data line-by-line through focused coding. We developed codes and code definitions in English, based on both English- and Spanish-language transcripts and observation notes. The two authors coded initial data separately, then compared, discussed, and reached an agreement if codes or emerging patterns matched or did not match [32]. After code testing and consensus coding, we applied codes to the data using qualitative analysis software, Dedoose (SocioCultural Research Consultants, LLC, Los Angeles, CA). We wrote memos to document patterns in the data and to comment on our methodological decisions. We re-sorted codes into themes and refined those themes by checking how well the coded extracts illustrated the themes.

## Results

As shown in Table 1, CHWs, program managers, and system-level participants represented government and non-governmental organizations, health care providers, health plans, academia, and foundations, that served urban areas or a combination of urban and surrounding rural areas. All stakeholders described the opportunities posed by plans for CHW certification in California but also warned of its unintended consequences. Four themes emerged among the three stakeholder groups (Table 2): 1) certification may enhance recognition of CHWs from health care providers and the community; 2) certification may offer more upward mobility and professional growth for CHWs; 3) certification may threaten CHW identity by pushing the CHW role into a clinical one and creating unintentional hierarchies (perhaps unintended) among certified and non-certified CHWs; and 4) certification may exclude some current or potential CHWs due to entry requirements.

**Table 1:** Characteristics of study participants

	**CHWs** **N (%)**	**Program Managers** **N (%)**	**Systems-level** **N (%)**
**Number of participants**	66 (100)	11 (100)	31 (100)
**Type of organization**			
Non-governmental organization^a^	22 (33)	7 (64)	11 (36)
Government^b^	5 (8)	1 (9)	10 (32)
Health plan	8 (12)	1 (9)	1 (3)
Health care provider^c^	17 (26)	2 (18)	3 (10)
Academia	0 (0)	0 (0)	4 (13)
Foundation	0 (0)	0 (0)	2 (6)
^d^Not defined	14 (21)	0 (0)	0 (0)
**Employment**			
Full-time	39 (59)	11 (100)	31 (100)
Part-time	5 (8)	0 (0)	0 (0)
Volunteer	8 (12)	0 (0)	0 (0)
^e^Not defined	14 (21)	0 (0)	0 (0)
**Language of Event**			
English	12 (18)	9 (82)	31 (100)
Spanish	31 (47)	0 (0)	0 (0)
^f^Bilingual	23 (35)	2 (18)	0 (0)
**Service area**			
Urban	39 (59)	7 (64)	7 (23)
Rural	5 (8)	1 (9)	0 (0)
Urban and rural	22 (33)	3 (27)	4 (13)
^g^Not defined	0 (0)	0 (0)	20 (64)
**Geographic Region**			
Northern California	1 (1)	1 (9)	4 (13)
Central California	3 (5)	1 (9)	1 (4)
Southern California	62 (94)	9 (82)	6 (19)
Statewide	0 (0)	0 (0)	14 (45)
National	0 (0)	0 (0)	6 (19)

^a^Non-governmental organizations (NGOs) included community-based organizations, coalitions, and advocacy and policy organizations

^b^Government included Local, state, and federal government

^c^Health care providers included individuals or organizations providing clinical services (e.g., federally qualified health centers, clinics, hospitals)

^d^Two focus groups with 14 total participants included a mixture of NGO and health care provider organizations but data were not collected to identify the type of organization for each individual participant

^e^Two focus groups with 14 total participants included a mixture of full-time, part-time, and volunteer CHWs but data were not collected to identify the employment status for each individual participant

^f^When both English and Spanish languages were spoken during an interview or focus group, the primary language of all participants was labeled “bilingual.”

^g^Stakeholder service area was labeled “not defined” when the stakeholders or stakeholder organization did not provide direct services to a specific geographic area

**Table 2:** Qualitative findings about CHW certification from CHW, program manager, and system-level participants

Common themes	Illustrative quote
**1) Certification may enhance recognition of CHWs from health care providers and the community**	
Certification could validate CHW work and provide recognition from health care providers	“[Health care providers] have better education, have better licensing, and better certifications. They don't see us like we belong. They make us feel like we're not important, we're not capable of doing what we do.”
Certification could help CHWs more effectively deliver information within their community by strengthening their credibility	“Creating a certification gives the person who is receiving the information the assurance that you are saying things correctly.”
**2) Certification may offer more upward mobility and professional growth for CHWs**	
Unified training and consensus about core CHW skills and knowledge could create the foundation for the professionalization of the CHW workforce in the state	“[CHWs are] not all the same depending on who trained them and what they’ve got. This uneven training could open the workforce to outside criticism, as employers may speculate: “‘You’re missing some basic skills, but yet you’ve been a CHW for 10 years.’”
Certification may result in career mobility	“CHWs will get hired and trained for one specific job, and then that job ends. Then they have to start from scratch and they just have whatever job they can find.”
Certification could lead to higher compensation and an established pay rate	“It’s not that money is important, I have 15 years of being a *promotora* and, believe me that all *promotoras*, yes, it is the love for the work, but we would be at a more recognized level [if we earned money.]”
**3) Certification may threaten CHW identity**	
Certification may dissolve the identity of CHWs and *promotores de salud* and push them into a more clinical role	“Institutions were under some pressure to get people into their program, and they were recruiting some people who might have been inappropriate for the work. They were certified, but they weren’t really CHWs. They were not from the community. They didn’t have anything in common with the community, but they had the training, and so they were entitled to call themselves certified CHWs, even though the community would probably look at them and say, ‘You’re not a CHW. You got a piece of paper, but you’re not a CHW. You’re not from here.’”
Tension between clinically focused and community-based roles could exacerbate identity differences between CHWs and *promotores de salud* in a state with no universal definition of CHWs	“We have never thought of a certification because we never thought of receiving money for our service. It’s always volunteer, we always do everything from the heart.”
Certification may create a hierarchy between certified and non-certified CHWs	“We have the entitlement piece where it’s like, ‘I’m certified and you’re not so I’m better at my job.’ That’s going to be a barrier, unfortunately.”
**4) Certification may exclude some current or potential CHWs due to entry requirements**	
Certification could exclude CHWs from working in their communities	“When you’re certifying people,” one CHW shared, “you’re limiting other groups of people getting the job done. You think it’s best for them, but when you get the certification you have to be literate, able to learn, be multi-tasking. It requires a little bit more steps that other people are not willing to do.”
Certification could exclude CHWs without legal residence	“They’re already facing racism, xenophobia, ICE [Immigration and Customs Enforcement] raids, and they don’t want to see this profession move along without remembering the unique contributions that they make in our state, and they’re worried particularly that certification will leave them behind.”
Certification could exclude CHWs with a history of incarceration	“Society [holds] stigma against people who are incarcerated,” noted one system-level participant, “There’s some really great people there, who have had this experience, who can turn it around and really help others and, that’s what makes them so successful.”
Certification could place more emphasis on state requirements than the vital social skills of connecting with communities	“[Training requirements] would actually knock a lot of people out of the workforce who actually demonstrated that they were great at connecting with [high-risk and high-needs patients].”

### Certification may enhance recognition of CHWs

Two themes emerged about CHW recognition. First, given that California’s CHWs lack a common training background, all types of participants believed that certification could validate CHW work and provide recognition from health care providers. CHWs described how certification could offer them recognition in a hierarchical health care system where certifications and licensures are necessary and valued. One CHW shared:

“[Health care providers] see us like, ‘Oh, a CHW.’ [Health care providers] have better education, have better licensing, and better certifications. They don't see us like we belong. They make us feel like we're not important, we're not capable of doing what we do. It's like they see us like [small] or the people that clean after me. They don't see we're capable of doing what we do. They just think that they're better than us because they have a title, and we don't have a title.”

CHWs expressed that their community experience, gained in their vital and intimate work with families in the community, is not understood or valued by many of the organizations they work for, especially in clinical settings. CHWs also shared their community experience positions them to identify community needs, communicate with families, and address social determinants of health better than most other health care providers. While all stakeholders commented on their desire for increased recognition of CHW skills, some commented that recognition from health care providers should not be dependent on certification. A system-level participant shared, “It’s a little sad that [certification] is how you see it’s necessary to get the respect of other people.”

All types of participants posed that educating health care providers about CHW contributions to care could be more impactful than certification. A few system-level participants believed that institutional racism and discrimination within the health care system discounts the CHW role and creates poor working conditions. “If you think about who the CHWs are,” a system-level participant shared, “they tend to be women…[and] women of color.” The participant described how the tendency to discount CHWs as “real health care providers” “justifies that they get paid less; they don’t get regular hours, shoddy training. It reinforces gender discrimination and racial discrimination.” System-level participants argued that certification could validate CHW work which may mitigate health care provider discrimination experienced by CHWs. Racism and discrimination towards CHWs impact the communities they serve because CHWs may be discouraged from facing racism in healthcare environments.

Second, CHWs speculated that certification could help them more effectively deliver information within their community by strengthening their credibility. A few CHWs recalled that some families they serve ask why they are qualified to provide education. A CHW shared, “Creating a certification gives the person who is receiving the information the assurance that you are saying things correctly.” CHWs shared that certification could give them the confidence to demonstrate their skills and training. CHWs believed that a certifying agency would have more weight with the families they serve and provide confidence in the services provided. A CHW shared, “We don’t have that support of saying, ‘I have this certification, I know what I do, please pay attention to me.’ We still don’t have that support of being trained.” CHWs suggested these questions about credibility undermines their training and expertise to deliver health education in their communities.

### Certification may offer more upward mobility and professional growth for CHWs

Three themes emerged about upward mobility and professional growth. First, all types of participants overwhelmingly agreed that unified training and consensus about core CHW skills and knowledge could create the foundation for the professionalization of the CHW workforce in the state. “[CHWs are] not all the same depending on who trained them and what they’ve got,” commented one system-level participant. This uneven training, continued the participant, could open the workforce to outside criticism, as employers may speculate: “‘You’re missing some basic skills, but yet you’ve been a CHW for 10 years.’” CHWs suggested that this continued failure to unify training and skills through certification could have negative effects for the families with whom they work, as some CHWs receive structured training programs and mentorship while other CHWs receive piecemeal trainings.

Second, all types of participants believed that CHW certification may result in career mobility. CHWs welcomed the opportunity that certification would afford them to grow in their careers. Certification could set boundaries for a workforce that other health professionals may be unfamiliar with and distinguish their work from health care providers with areas of overlapping expertise, such as social workers. Further, certification could establish a career ladder or series of positions that would enable them to advance professionally with increasing experience. System-level participants commented that, in California, CHW job transitions are mostly lateral. A system-level participant shared, “CHWs will get hired and trained for one specific job, and then that job ends. Then they have to start from scratch and they just have whatever job they can find.” Participants believed that certification could generate a stronger field of positions when CHWs sought work transitions. Professionalizing the workforce could enable CHWs to leverage better pay, better positions, and career mobility through recognition of their skills.

In observations, some stakeholders showed concern that a CHW career ladder would threaten the roles of health care providers. Some health care provider advocacy organizations feared that CHW certification would lead to “scope creep,” with CHWs potentially infringing on their professional scope of practice, diminishing their current clinical roles and clinical support for their licensed professions. Conversely, some system-level participants noted the hypocrisy embedded in this reaction to building CHW career mobility: “Other professions forget that established professions objected to them on the way up. Nurses had to struggle for…professionalization. Midwives did. Health educators did. MSWs [social workers] did. But they’re really quick to turn around and say, ‘But I don’t know about you.’”

Last, all participants were united in their belief that CHW certification could lead to higher compensation and an established pay rate. CHWs felt that the potential for increased pay from certification could result from better recognition of their value, which is modestly remunerated or, in the case of *promotores de salud*, not compensated. Uncompensated *promotores de salud* clarified that, though pay was not the motivation for their work, certification may enable them to earn money: “It’s not that money is important, I have 15 years of being a *promotora* and, believe me that all *promotoras*, yes, it is the love for the work, but we would be at a more recognized level [if we earned money.]” Program managers and system-level participants believed that certification may facilitate the payment of CHW services by insurers, allowing payers clarity of the CHW role in care. A system-level participant shared that payers’ confusion about CHW roles was a barrier to supporting their role in care: “They don’t want to pay for something that they’re not clear about.” Certification could enable sustainable financing models from federal payers, such as Medicaid, that include strict regulations on spending categories and service providers. Without certification, noted a system-level participant, “I find it hard to imagine that CHWs’ time is going to be reimbursed in the model and structure of the health care setting that we have now. These are federal dollars, there’s rules.” Yet others cautioned that certification alone will not guarantee Medicaid financing. Another system-level participant suggested that Medicaid financing for CHW services relies more on the advocacy of CHW champions, state-specific legislation, and payer systems rather than singly on certification.

### Certification may threaten CHW identity

Three themes related to CHW identity emerged. First, all participants expressed fears that certification may dissolve the identity of CHWs and *promotores de salud* and push them into a more clinical role, further away from the historical community-centered role. Some CHWs and program managers were concerned that certification could attract people without a commitment to the community-centered CHW role, but instead sought to use certification as a “stepping stone” into other health care positions, such as social work or nursing. Some system-level participants commented on evidence from states that have implemented CHW certification that validated these fears. A system-level participant who worked on certification outside California shared,

“Institutions were under some pressure to get people into their program, and they were recruiting some people who might have been inappropriate for the work. They were certified, but they weren’t really CHWs. They were not from the community. They didn’t have anything in common with the community, but they had the training, and so they were entitled to call themselves certified CHWs, even though the community would probably look at them and say, ‘You’re not a CHW. You got a piece of paper, but you’re not a CHW. You’re not from here.’”

A system-level participant reflected that instructors in CHW certification programs could distinguish who in their class possessed “real CHW” qualities, and others who may eventually leave the profession, believing that individuals were motivated by the certification rather than serving their community.

As California CHWs represent distinct ideologies of care, program managers and system-level participants were wary that certification could push more CHWs into clinical roles. Because clinical organizations place more emphasis on certifications and degrees, participants anticipated all CHWs employed by these organizations would pursue certification. Since certifications are not required by community-based organizations, participants believed that community-based CHWs and *promotores de salud* may not pursue certification. All types of participants worried that certification could produce a disparity between clinical and community-based CHWs or *promotores de salud* due to the demands of their organizations. Program managers and system-level participants also feared that the clinical orientation of certification would transform CHWs to fit the health care system and undermine *promotores de salud* historic emphasis on impacting the social determinants of health through community-based work.

Second, system-level participants expressed concern that the tension between clinically focused and community-based roles could exacerbate identity differences between CHWs and *promotores de salud* in a state with no universal definition of CHWs. A system-level participant described how funding sources already separated *promotores de salud* and CHWs. *Promotores de salud* have historically served Latinx communities, working at the intersection of health and social justice, predominantly as volunteers. CHWs were more recently employed by health departments, clinics, and hospitals, driven by single source funding opportunities. (Other stakeholders described CHWs and *promotores de salud* as interchangeable titles but serving different populations.) Yet *promotores de salud* participants were less likely to consider certification relevant for their work, citing the intrinsic motivation for their work. A *promotor de salud* shared, “We have never thought of a certification because we never thought of receiving money for our service. It’s always volunteer, we always do everything from the heart.” *Promotores de salud,* describing their work as service rather than a professional career, asserted that certification was not relevant to their motivation to serve their communities and should not dictate who may work as a CHW. For community-based CHWs, *promotores de salud*, and program managers, certification does not challenge the identity of the CHW role. Their identity goes beyond a certification process because their intrinsic motivation to serve their community defines their CHW role instead of skills identified by a certifying authority. A CHW shared, “If you don’t have that certificate, that doesn’t mean you’re not a CHW.”

Third, CHWs and program managers anticipated that certification may create a hierarchy between certified and non-certified CHWs, in which certified CHWs may have an unfair advantage for employment and feel superior to non-certified peers because of the certification, exacerbating other existing inequalities between CHWs in employment type (e.g., paid or volunteer) and work setting (e.g., community or clinic). A program manager shared, “We have the entitlement piece where it’s like, ‘I’m certified and you’re not so I’m better at my job.’ That’s going to be a barrier, unfortunately.” Still, system-level participants from states that have implemented CHW certification shared they have not directly observed a hierarchy among CHWs that was feared for California.

### Certification may exclude some current or potential CHWs

Four themes emerged about exclusion. First, nearly all participants were concerned that instituting certification could exclude CHWs from working in their communities- whether they were existing CHWs or those considering the career path. “When you’re certifying people,” one CHW shared, “you’re limiting other groups of people getting the job done. You think it’s best for them, but when you get the certification you have to be literate, able to learn, be multi-tasking. It requires a little bit more steps that other people are not willing to do.” The very qualities that make CHWs so effective in providing culturally and linguistically appropriate care can also be vulnerabilities that certifying authorities may not value or recognize. A CHW shared their fear of excluding people with crucial shared experiences but little formal education, commenting, “Moms who cannot read or write will not have the chance to be *promotores*.” All participants believed that certification, as a complex process overseen by the state, posed multiple barriers to a workforce largely comprised of women of color from marginalized, multi-lingual communities. Potential barriers to obtaining certification mentioned were numerous, such as literacy level, training costs, availability of certification in languages other than English, education requirements, and legal residence status. Further, participants believed the many CHWs who lived in communities with geographic and technological barriers to accessing training sites and materials risked being shut out of certification entirely.

Second, all participants were concerned about the implications of certification for undocumented CHWs in California since most certification processes require legal residence documentation. System-level participants worried that certification could prevent undocumented CHWs from continuing to work in immigrant communities. “They’re already facing racism, xenophobia, ICE [Immigration and Customs Enforcement] raids, and they don’t want to see this profession move along without remembering the unique contributions that they make in our state,” explained one participant. “And they’re worried particularly that certification will leave them behind.”

Third, some CHWs who experienced prior incarceration worked with individuals to navigate the psychosocial and structural struggles after “coming home” from prison. All types of participants feared that certification requirements for a criminal background check could exclude these CHWs with prior felonies. “Society [holds] stigma against people who are incarcerated,” noted one system-level participant, “There’s some really great people there, who have had this experience, who can turn it around and really help others and, that’s what makes them so successful.” Excluding CHWs with felony convictions could eliminate a vital point of support for an extremely vulnerable population.

Last, many feared that certification could exclude those CHWs who are most effective in their communities by placing more emphasis on state requirements than the vital social skills of connecting with communities. Participants questioned the extent to which certification could assess whether CHWs are equipped to do their work and cautioned that relationship-building skills and lived experience are difficult to evaluate through a certification system. A system-level participant shared that such training requirements “would actually knock a lot of people out of the workforce who actually demonstrated that they were great at connecting with [high-risk and high-needs patients].” Participants believed that state certification requirements could overshadow what CHWs do best: developing trusting relationships within their communities and advocating for positive change. A system-level participant illustrated this concept with a *promotores de salud* motto: “Don’t change who we are but give us a chance,” emphasizing that workforce standardization could inevitably alter the qualities that make CHWs and *promotores de salud* effective.

## Discussion

We set out to understand diverse stakeholder perspectives on CHW certification as California considers certification’s capacity to advance or potentially exclude members of this critical workforce. This study was strengthened by CHW perspectives about certification and provided crucial insights about the diverse membership of CHWs. Peer-reviewed CHW certification literature is limited, and existing scholarship largely omits CHW perspectives on setting their own workforce standards [19], despite their expertise and deep knowledge of the organizational and systemic levers necessary to implement community programs. Echoing literature on certification [17, 22, 33–37], we found conflicting perspectives within and between all stakeholders groups on certification. Our findings confirm some findings from a 2021 study that explored perspectives on certification from seven states that have implemented or considered implementing certification [22] (e.g., CHW participation in decision making, concerns about certification requirements excluding CHWs). In this study, some participants affirmed the financial and career opportunities offered by certification, while others feared it may inadvertently exclude vulnerable CHWs and the communities they serve and downplay advocacy at the heart of CHW identity. Participants also highlighted how certification does not address all challenges related to integrating CHWs into health care systems.

Our findings underline how California CHWs desire opportunities to grow. We confirmed that CHWs view certification as an opportunity to increase compensation and to build career opportunities; not just through expanding the capacity of organizations to work with CHWs, but also by building a career ladder [38]. Participants agreed certification may bring health care provider and community recognition and increase the demand for CHW services. Our findings confirmed literature asserting that health care providers feel more confident when CHWs are certified because they can ensure a standard of care [39].

At the same time, certification provoked fears across many participants that CHWs will transform into a more clinical role, potentially diminishing the tradition of advocacy, social justice, and community connection, echoing existing literature [23, 33, 40, 41]. CHW participants emphasized their skill in addressing the social determinants of health and noted that this essential contribution might be undermined if CHW work were restricted within a medical model of health. By “professionalizing” the CHW workforce, certification could threaten the qualities that make CHWs effective, such as gaining community trust [38]. *Promotores de salud* stakeholders fear that certification washes away the “essence” of *promotores* by attracting people without “the heart” for the work [42]. A broader group of participants doubted whether certification could cultivate or measure the relational skills that help constitute this “heart.”

Existing research suggests these fears may be warranted. A Zambian study found that focusing on skills or career incentives, rather than community service, displaced CHWs with desirable social connections and lessened the quality of services they provided [43]. Our findings also demonstrate CHWs’ skepticism that individuals will use certification as a “stepping stone” to other health professions, potentially resulting in high turnover of the CHW workforce. A national survey of CHW certification programs confirms this prediction- finding that many CHWs, after attending community college certification training programs, later advanced to nursing and social work professions [24].

Participants in this study also stressed the limits of certification as a tool to advance the CHW workforce. Our findings echo existing literature asserting that certification does not guarantee employment or Medicaid financing for CHWs [38, 39, 44, 45] but continues to be a motivation for financial sustainability for the workforce [22]. This is a timely finding as California’s Department of Health Care Services (the agency responsible for administering Medicaid in California) has taken federal actions to finance CHW-delivered services for Medi-Cal members and California’s managed care organizations have begun integrating CHWs into their standard of care [20, 30, 46, 47]. While some participants appreciated the increase in recognition that certification may offer, other studies show that funding streams and return on investment have been identified by employers as the most important factors in whether to hire CHWs [48]. While our participants hoped that certification would affirm the quality of care delivered by CHWs in their communities and to their employers, there is not yet conclusive evidence CHW training and certification programs ensure quality of services delivered, as there have been limited evaluations in states to date [24].

One of the key contributions of CHWs and *promotores de salud* in this study was that they identified their uncertainties that certification will engender unintentional hierarchies: both between clinical and community-based CHWs, and between CHWs and *promotores de salud*. Over time, employers may prefer certified CHWs since they could ensure a standard skill set and knowledge base [38], unofficially making the certification “required” [49]. Since community-based CHWs and *promotores de salud* have little need for certification in their communities, their work—which already receives less funding—may become further marginalized. We fear certification may result in fewer community-based CHWs and *promotores de salud* working in marginalized communities, ultimately diminishing access to services delivered by culturally and linguistically appropriate professionals who embody community trust.

Our study highlights how racism and discrimination within the health care system may have obstructed the advancement of the CHW workforce into health care systems, a theme unacknowledged in previous CHW research. This insight adds evidence of documented racism towards paraprofessionals, particularly among structurally similar positions like certified nursing assistants who experience institutional racism, cultural insensitivity, and discrimination from supervisors and coworkers [50–54]. While public health and health care institutions have supported the concept that CHWs are key to diversifying the health care and public health workforces [55], their recommendations have not acknowledged that racism within the health care system remains a critical barrier to this diversification.

To the best of our knowledge, this is the first study to describe perspectives of CHW certification from CHW, *promotores de salud*, program manager, and system-level stakeholders using existing certification models. However, this study is not without limitations. The CHW participants were almost exclusively from Southern California. CHWs from Northern California may have had different perspectives on certification. There were two focus groups for which employment status and organization type data were not collected. We do not have basic demographic information for participants (e.g., age, gender, race/ethnicity). One type of CHW, known as a Community Health Representative (CHR), works with Native American clients as trusted members of their tribal communities and connects them to health care and social services [56]. Scheduling logistics limited our ability to reach CHR informants. Patient perspectives were also not included in this study. All these issues limit the conclusions we can draw about an entire state.

To address the concerns identified by participants, we suggest a certification process where CHWs may choose whether they want to become certified and consists of multiple, accessible pathways to certification: a training pathway (where new CHWs learn required skills and knowledge) and a work experience pathway (existing CHWs may certify based on amount and type of experience) [19, 57].To date, nearly every state that has certification offers existing CHWs an opportunity to certify based on their work experience [58]. Approximately 15 states have implemented certification based on training [44, 46, 58]. Echoing a recent peer-reviewed study of certification, this certification approach would limit requirements of formal education, English-language proficiency, history of incarceration, and legal residence status [19, 22, 57, 59, 60] to continue to foster the diversity that is a key feature of an effective CHW workforce. Most importantly, this approach respects *promotores de salud* and other CHWs that may not need certification for their work by not mandating certification and acknowledging their contributions [19].

When designing CHW certification, stakeholders must position CHWs as key decision makers. Our study echoes existing literature identifying CHWs as essential and best equipped to lead workforce discussions because they can better anticipate the needs and aspirations of their workforce and should make up at least half of the decision makers [22, 61]. We anticipate if CHWs are not included in the decision making, certification could reflect a process that may unintentionally exclude workforce members and limit services to communities in need.

## Conclusion

Considering the high stakes posed by certification, we appraised its potential through evaluating the perspectives of diverse stakeholders who represented various CHW types and organizational roles. Certification may increase CHWs’ legitimacy within clinical organizations and communities by expanding recognition of their contributions and increasing their professional security. Yet certification poses genuine challenges to delivering care by CHWs that value relationships, prioritize cultivating the social determinants of health outside clinical settings, and favor connection with vulnerable communities largely excluded from formal systems of education. Our findings affirm neither certification nor its absence, but instead a nuanced path ahead that mixes opportunities with continued reflections on how the CHW workforce can be supported for what it does best. Above all, CHW participation in designing certification processes is essential to protect “the heart” and diversity of the workforce.

## Data Availability

Participants did not give consent for transcripts to be published in public repository. Codebook available upon request.

## Abbreviations

CHW: Community health worker
NGO: Non-governmental organization
ICE: Immigration and Customs Enforcement
CHR: Community Health Representative
